# *Pycnoporus cinnabarinus* glyoxal oxidases display differential catalytic efficiencies on 5-hydroxymethylfurfural and its oxidized derivatives

**DOI:** 10.1186/s40694-019-0067-8

**Published:** 2019-04-01

**Authors:** Marianne Daou, Bassem Yassine, Saowanee Wikee, Eric Record, Françoise Duprat, Emmanuel Bertrand, Craig B. Faulds

**Affiliations:** 1 INRA, UMR1163 Biodiversité et Biotechnologie Fongiques (BBF), Aix Marseille Université, 13009 Marseille, France; 2 CNRS, Centrale Marseille, Aix-Marseille Université, M2P2, Marseille, France

**Keywords:** 5-Hydroxymethylfurfural, Bio-catalysis, Glyoxal oxidase, 2,5-Furancarboxylic acid, Furan derivatives

## Abstract

**Background:**

5-Hydroxymethylfurfural (HMF), a major residual component of a lignocellulosic bio-refinery process, can be transformed into fundamental building blocks for green chemistry via oxidation. While chemical methods are well established, interest is also being directed into the enzymatic oxidation of HMF into the bio-plastic precursor 2,5-furandicarboxylic acid (FDCA).

**Results:**

We demonstrate that three glyoxal oxidases (*Pci*GLOX) isoenzymes from the Basidiomycete fungus *Pycnoporus cinnabarinus* were able to oxidize HMF, with *Pci*GLOX2 and *Pci*GLOX3 being the most efficient. The major reaction product obtained with the three isoenzymes was 5-hydroxymethyl-2-furancarboxylic (HMFCA), a precursor in polyesters and pharmaceuticals production, and very little subsequent conversion of this compound was observed. However, small concentrations of FDCA, a substitute for terephthalic acid in the production of polyesters, were also obtained. The oxidation of HMF was significantly boosted in the presence of catalase for *Pci*GLOX2, leading to 70% HMFCA yield. The highest conversion percentages were observed on 2,5-furandicarboxaldehyde (DFF), a minor product from the reaction of *Pci*GLOX on HMF. To bypass HMFCA accumulation and exploit the efficiency of *Pci*GLOX in oxidizing DFF and 5-formyl-2-furan carboxylic acid (FFCA) towards FDCA production, HMF was oxidized in a cascade reaction with an aryl alcohol oxidase (*Uma*AAO). After 2 h of reaction, *Uma*AAO completely oxidized HMF to DFF and further to FFCA, with FDCA only being detected when *Pci*GLOX3 was added to the reaction. The maximum yield of 16% FDCA was obtained 24 h after the addition of *Pci*GLOX3 in the presence of catalase.

**Conclusions:**

At least two conversion pathways for HMF oxidation can be considered for *Pci*GLOX; however, the highest selectivity was seen towards the production of the valuable polyester precursor HMFCA. The three isoenzymes showed differences in their catalytic efficiencies and substrate specificities when reacted with HMF derivatives.

**Electronic supplementary material:**

The online version of this article (10.1186/s40694-019-0067-8) contains supplementary material, which is available to authorized users.

## Background

With the growing concerns about the depleting supply of fossil fuel and the global problem of climate change, the demands for sustainable substitutes for petroleum-based products are increasing. Being one of the most abundant renewable natural materials on earth, plants have become a major candidate for this role, especially those species that yield low-cost residues from agro-industrial processing. Approximately 75% of annual production of agro-industrial residues is in the form of carbohydrates, making this material highly exploitable [1]. Efforts are being directed for the conversion of plant carbohydrates into valuable chemicals and fuels. Among the valuable platform chemicals that can be obtained from biomass carbohydrates are 5-hydroxymethylfurfural (HMF) and furfural [2], produced by triple dehydration of hexoses [3] and acid hydrolysis of pentosans, respectively [4].

HMF is particularly important due to its biodegradability and its versatility as a precursor for a wide selection of furan-based products, and has been recognized as top value-added molecule in biotechnology [5]. Important molecules derived from HMF include dimethylfuran, levulinic acid, 2,5-furandicarboxylic acid (FDCA), 2,5-diformylfuran (DFF), 3,5-dihydroxymethylfuran, 5-hydroxy-4-keto-pentenoic acid and 5-hydroxymethyl-2-furancarboxylic acid (HMFCA) [6]. DFF is a stable derivative of HMF. This molecule is considered important for the synthesis of pharmaceutical compounds [7], antifungal products [8], electroconductors [9] and polymeric materials [10]. Another important product from the oxidation of the aldehyde group of HMF is HMFCA. This is particularly important for the production of polyesters [11] and interleukin inhibitors [12], and was found to have in vivo antitumor activity against Sarcoma 180 cells [13]. Furan-based derivatives also include FDCA, which was considered among the 12 most promising sugar-based molecules for the production of bio-based material [14]. FDCA is particularly important because it can replace fossil-based terephthalic acid for the production of bio-based polyesters [15]. Chemically, HMF derivatives are produced using metals as catalysts under extreme conditions such as high temperature, high pressure and in the presence of toxic solvents. The development of bio-catalytic processes is therefore advantageous since enzymes work under mild conditions, which can reduce the energy required and the production cost.

**Fig. 1:**
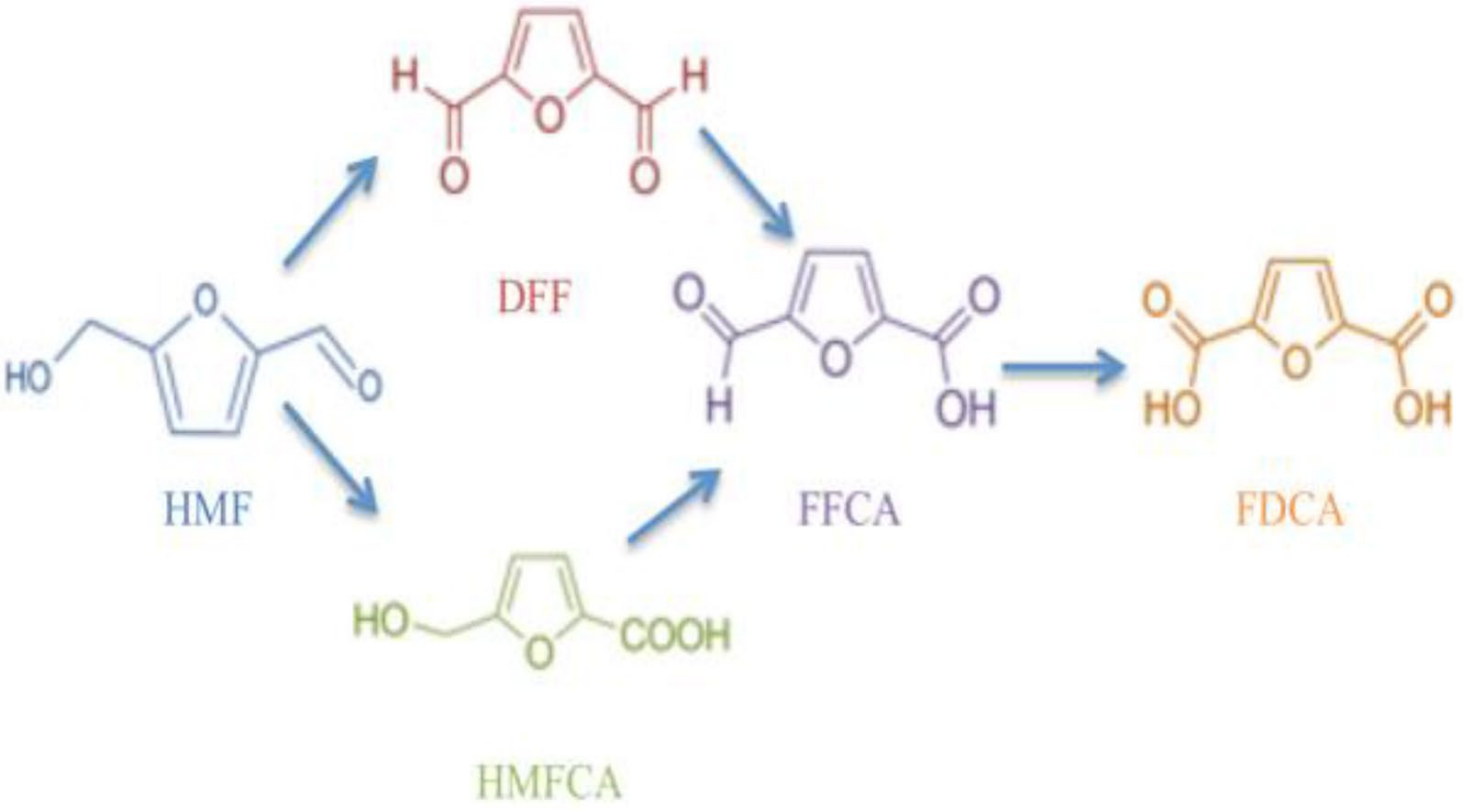
The oxidation reaction pathways of HMF to FDCA (Carro et al. [18])

Among the enzymes that were previously tested and used for the oxidation of HMF are three alcohol oxidases (EC 1.1.3.13) and one galactose oxidase (GAO; EC 1.1.3.9) that have been reported to oxidize HMF leading to the formation of DFF as the sole product following the reaction presented in Fig. 1 [16]. Synthesis of HMFCA by bio-catalysis remained limited due to the need of high reaction selectivity in order to oxidize the aldehyde group and leave the hydroxyl group intact [17]. Xanthine oxidase (EC 1.17.3.2) from *Escherichia coli* was used for the selective oxidation of HMF to HMFCA [16]. Oxidative conversion of HMF to FDCA was also performed following a two-steps reaction using fungal aryl-alcohol oxidase (AAO; EC 1.1.3.7) and an unspecific heme peroxygenase (EC 1.11.2.1) [18]. Full HMF conversion with high FDCA yields was also achieved using an FAD-dependent oxidase, named HMF oxidase (HMFO; EC 1.1.3.47) [19, 20].

Although HMF derivatives are highly important, limited examples describing the synthesis of these compounds via enzymatic reactions were previously described and the pursuit for new enzymes that can be involved in this process is still highly interesting. Promising candidates are enzymes with versatile substrate specificity able to perform the successive oxidation steps required in the process by recognizing HMF and its derivatives as substrates.

Glyoxal oxidases (GLOX; EC 1.2.3.15) are metalloenzymes containing one copper metal ion, and belong to a class of enzymes called radical copper oxidases [21]. These enzymes have been grouped within the AA5 family in the CAZymes (Carbohydrate Active Enzymes database; http://www.cazy.org/) classification system [22, 23]. GLOX catalyzes the oxidation of aldehydes to their corresponding carboxylic acids, generating simultaneously hydrogen peroxide (H_2_O_2_) [24, 25]. This enzyme was first isolated in ligninolytic cultures of the white-rot Basidiomycete *Phanerochaete chrysosporium* under restricted nitrogen supply, suggesting a role in lignin modification and peroxidase H_2_O_2_-dependent action [26]. Subsequent sequencing and analysis of the *P. chrysosporium* genome has indicated the presence of only one gene encoding GLOX in this fungus [27]. The genome of another Polyporale fungus, *Pycnoporus cinnabarinus* [28], contains 3 *glox* genes. In vitro characterization of these GLOX revealed their ability to act on a broad range of substrates including toxic and inhibitory aldehydes [24, 25]. In addition, GLOX were able to recognize and oxidize the alcohol group of glycerol extending further the specificity range of these enzymes [25, 29]. Although the utilization of GLOX for biotechnological applications had not previously been elaborated upon, the reactions catalyzed by this enzyme made it a promising candidate, especially for the oxidation of HMF and its derivatives.

In this work, the ability of two previously produced and characterized GLOX from *P. cinnabarinus* strain BRFM 137 (*Pci*GLOX1 and *Pci*GLOX2) [25] and a third isoform from this fungus (*Pci*GLOX3) to oxidize HMF and its derivatives was investigated.

## Results

### *Pci*GLOX3 characterization

**Fig. 2:**
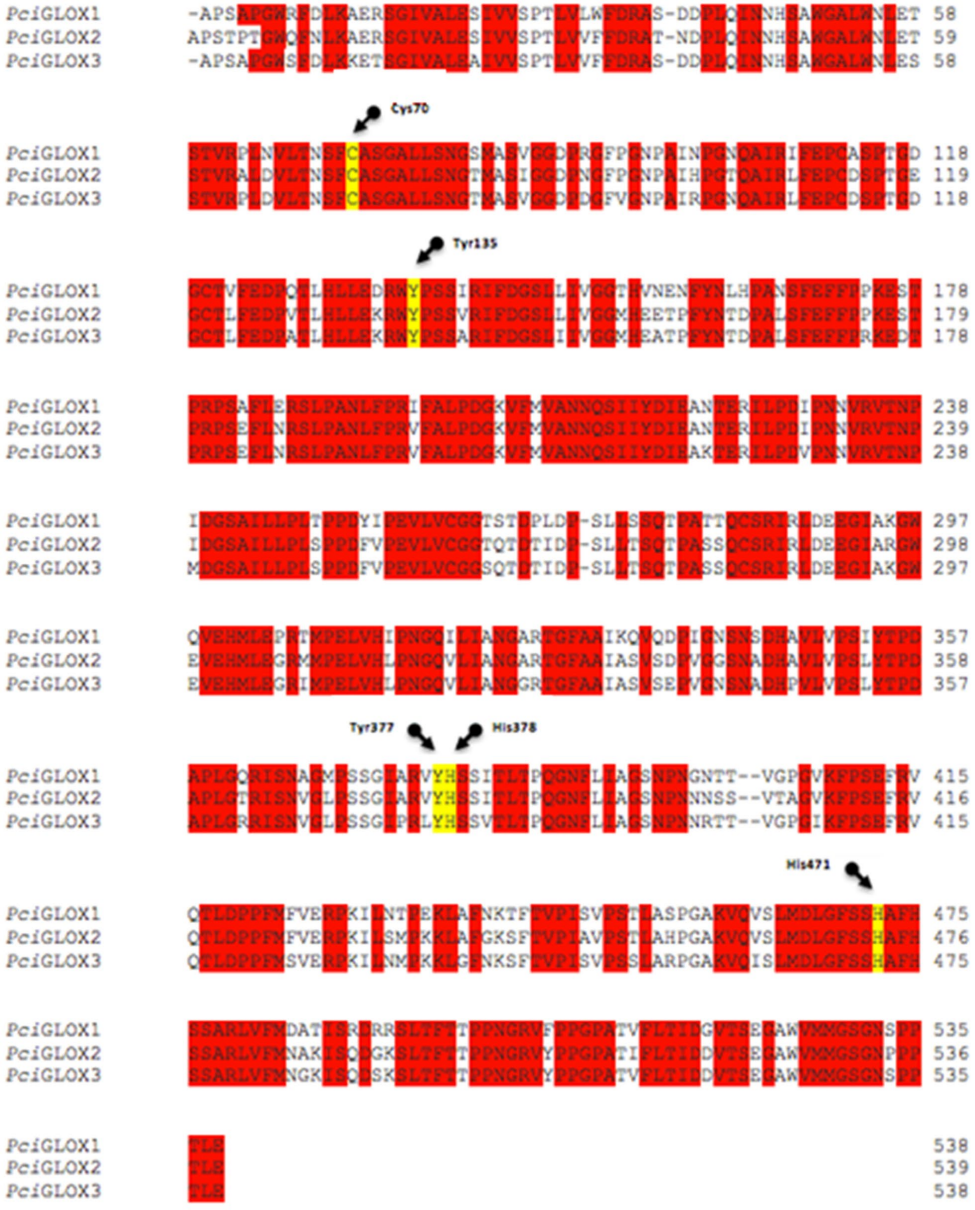
Custal W alignment of *P. cinnarbarinus* GLOX proteins. Identical residues are highlighted in red. Arrows indicate residues essential for catalysis

*Pci*GLOX3 shared 84% and 89% protein sequence identity with *Pci*GLOX1 and *Pci*GLOX2, respectively, and the amino acids at the active site were highly conserved (Fig. 2). *Pci*GLOX3 was heterologously produced in *Aspergillus niger* and purified. The enzyme showed a generally lower activity on the tested substrates compared to the other two *Pci*GLOX enzymes, however, the specificity range for all three isoenzymes was similar except in the case of glycerol, which was only oxidized by *Pci*GLOX2 and *Pci*GLOX3 (Table 1). One major difference observed was that highest activity detected on formaldehyde for *Pci*GLOX3 while the two other enzymes were most active on glyoxylic acid. Differences in the kinetic constants were also observed for *Pci*GLOX3 that showed the highest specificity and catalytic efficiency on glycerol compared to the other enzymes (Table 2). In addition, *Pci*GLOX3 was less efficient in oxidizing glyoxylic acid compared to the two other GLOX.

**Table 1:** Substrate specificity of *Pci*GLOX3 compared to previously characterized *Pci*GLOX1 and *Pci*GLOX2 (Daou et al. [25])

Substrate (10 mM)	Activity (nkat/mg)		
	*Pci*GLOX1	*Pci*GLOX2	*Pci*GLOX3
Methyl glyoxal	814	291	90
Glyoxal	392	163	68
Glyoxylic acid	1562	384	59
3-Phenylpropionaldehyde	10	135	51
Formaldehyde	50	202	133
DL-glyceraldehyde	11	66	26
Dihydroxyacetone	108	157	64
Glycerol	2	61	88
2,4-Dimethoxybenzaldehyde	ND	2	ND
Veratraldehyde	ND	ND	ND
4-Hydroxybenzaldehyde	ND	ND	ND
Phenyl glyoxilic acid	ND	ND	ND
Formic acid	ND	ND	ND
d-glucose	ND	ND	ND
d-galactose	ND	ND	ND
d-xylose	ND	ND	ND
Methanol	ND	ND	ND

ND, activity not detected under these assay conditions

**Table 2:** Kinetic constants of *Pci*GLOX3 on Methylglyoxal, glyoxal, glyoxylic acid and glycerol compared to *Pci*GLOX1 and *Pci*GLOX2 (Daou et al. [25])

Substrate	*Pci*GLOX1		*Pci*GLOX2		*Pci*GLOX3	
	*K*_m_ (mM)	*K*_cat_/*K*_m_ (s^−1^ mM)	*K*_m_ (mM)	*K*_cat_/*K*_m_ (s^−1^ mM)	*K*_m_ (mM)	*K*_cat_/*K*_m_ (s^−1^ mM)
Methylglyoxal	1.3	58.4	0.2	7	0.1	75.8
Glyoxal	13.1	6.3	2.2	0.6	0.7	11.2
Glyoxylic acid	0.08	2136.3	0.1	17	0.5	15.3
Glycerol	660.5	0.04	9.4	0.06	5.5	1.4

### Enzyme stability under the conditions of reaction

**Fig. 3:**
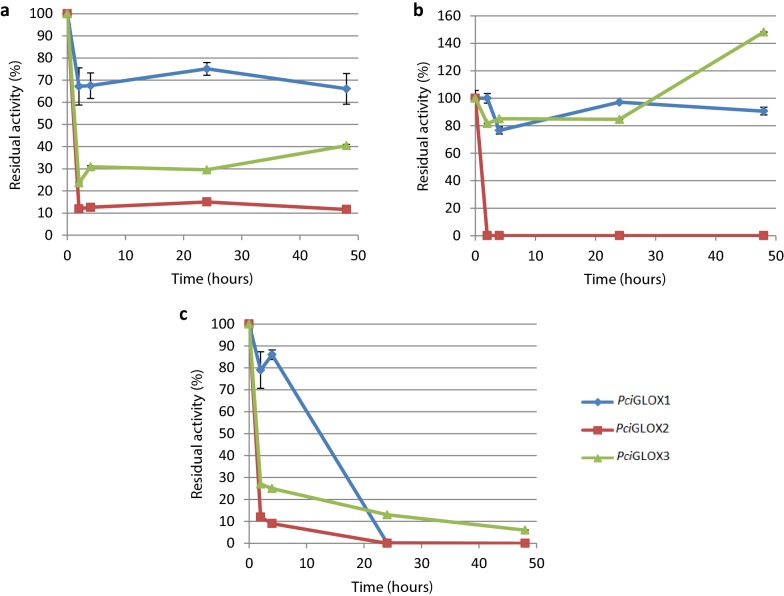
Residual activity of the three *Pci*GLOX after incubation at 30 °C and 800 rpm shaking in 50 mM tartrate buffer pH6 in the absence (**a**) and presence (**b**) of 3 mM HMF, and in the presence of 3 mM HMFCA (**c**). The residual activity was calculated as a percentage of the activity before incubation. Bars represent standard deviation

The standard reaction conditions for the oxidation of HMF and its derivatives were chosen based on the stability and performance of the enzymes under these conditions. The optimal pH was 6 and the enzymes were active in dimethylsuccinate and tartrate buffers [25]. The reactions were performed in tartrate buffer, as dimethylsuccinate could not be resolved from the substrates and expected products of the enzymatic reaction in the HPLC analysis. The stability of *Pci*GLOX enzymes under these conditions and over a prolonged period of time were assessed. *Pci*GLOX2 was the least stable of the three isoenzymes and lost around 90% of its activity after 2 h of incubation (Fig. 3a). On the other hand, *Pci*GLOX1 and *Pci*GLOX3 retained 70% and 40% of their activities after 48 h of incubation, respectively. The stability of the three enzymes was also assessed in the presence of 3 mM HMF. In the presence of this substrate, no activity was detected for *Pci*GLOX2 after 2 h of incubation (Fig. 3b). On the other hand, the activities of *Pci*GLOX1 and *Pci*GLOX3 were relatively stable with *Pci*GLOX3 showing increased residual activity after 48 h. The stability of the three *Pci*GLOX enzymes was further investigated in the presence of 3 mM HMFCA, which is the major theoretically expected product of the reaction on HMF. In the presence of this molecule, the significant loss in activity after 24 h of incubation was comparable between the three isoforms (Fig. 3c). Horseradish peroxidase (HRP) was highly stable under the tested conditions (Additional file 1).

### Stability towards H_2_O_2_

**Fig. 4:**
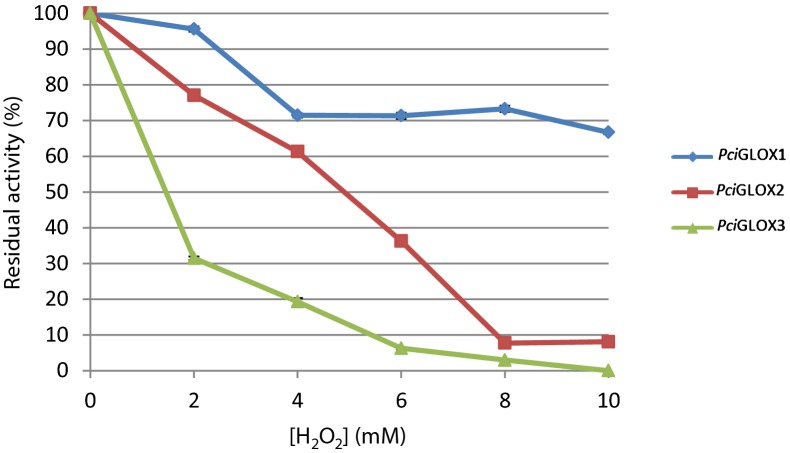
Stability of the three *Pci*GLOX in increasing concentrations of hydrogen peroxide after an incubation period of 24 h. Bars represent standard deviation

The stability of the three *Pci*GLOX isoenzymes was investigated in the presence of varying concentrations of H_2_O_2_. *Pci*GLOX1 was the most stable enzyme in the presence of this reaction product, retaining 60% of its activity when incubated with 10 mM H_2_O_2_ for 24 h (Fig. 4). Although more stable than *Pci*GLOX3, *Pci*GLOX2 significantly lost activity with increasing concentrations of H_2_O_2_ and lost more than 95% of its activity after incubation in the presence of 8 mM H_2_O_2_. *Pci*GLOX3 on the other hand lost 60% of its activity with only 2 mM H_2_O_2_ and was completely inactive with 10 mM H_2_O_2_ after 24 h of incubation.

### Oxidation of HMF and its derivatives by *Pci*GLOX

**Fig. 5:**
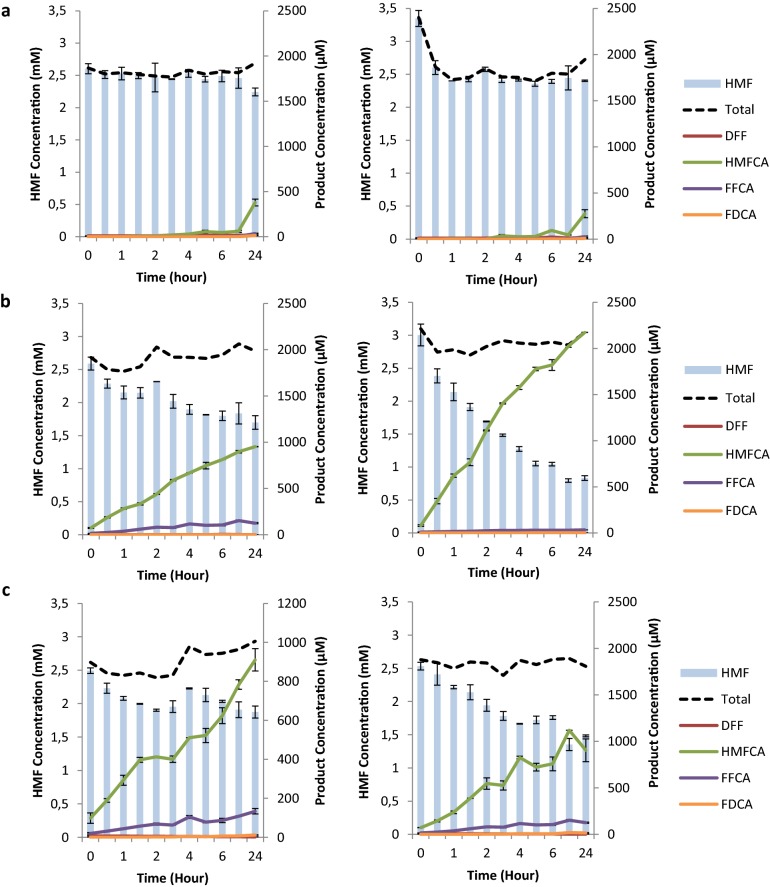
HMF oxidation reaction follow-up over time in the absence (left) and presence (right) of catalase by **a** *Pci*GLOX1, **b** *Pci*GLOX2 and **c** *Pci*GLOX3. Bars represent standard deviation

The three *Pci*GLOX isoenzymes were found to be active on HMF and this became evident by the decrease in HMF concentration and the appearance of the oxidation products over time (Fig. 5, Additional file 2). Although the catalytic efficiencies of the three enzymes were comparable on HMF (Table 3), the conversion percentage of this substrate varied between the *Pci*GLOX enzymes (14% for *Pci*GLOX1, 34% for *Pci*GLOX2 and 28% for *Pci*GLOX3). In addition, the three isoenzymes noticeably differed in their products’ patterns.

**Table 3:** Kinetic parameters for the oxidation of HMF, DFF, and FFCA by the three *Pci*GLOX isoenzymes. Standard deviations are presented as plus–minus values

Substrate	*Pci*GLOX1	*Pci*GLOX2	*Pci*GLOX3
*HMF*			
*K*_m_ (mM)	15.66 ± 2.35	5.87 ± 2.04	6.35 ± 1.32
*K*_cat_ (s^−1^)	1.59 ± 0.12	0.56 ± 0.09	0.75 ± 0.07
*K*_cat_/*K*_m_ (s^−1^ M^−1^)	101.66 ± 0.01	96.04 ± 0.01	118.35 ± 0.01
*DFF*			
*K*_m_ (mM)	4.38 ± 0.1	0.21 ± 0.04	0.18 ± 0.05
*K*_cat_ (s^−1^)	0.54 ± 0.24	4.80 ± 0.24	1.28 ± 0.09
*K*_cat_/*K*_m_ (s^−1^ M^−1^)	124.39 ± 0.01	23,403.66 ± 0.01	7267.72 ± 0.01
*FFCA*			
*K*_m_ (mM)	0.85 ± 0.14	1.40 ± 0.39	0.61 ± 0.58
*K*_cat_ (s^−1^)	0.03 ± 0.01	2.02 ± 0.03	0.04 ± 0.01
*K*_cat_/*K*_m_ (s^−1^ M^−1^)	38.55 ± 0.01	1435.45 ± 0.01	72.03 ± 0.01

The major product of the reactions of the *Pci*GLOX isoenzymes on HMF was HMFCA with *Pci*GLOX2 and *Pci*GLOX3 producing the highest yields (39% and 41%, respectively; Fig. 5b, c). By adding catalase to the reaction of *Pci*GLOX2, the amount of produced HMFCA was considerably increased to 76% (Fig. 5b right). This increase was coupled to a significant boost in HMF conversion (p = 0.062).

Interestingly, the addition of catalase seemed to shift the reaction more towards the production of HMFCA and decreased the amount of 5-formyl-2-furan carboxylic acid (FFCA) produced from 5 to 0.7%. A weak activity-enhancing effect in the presence of catalase was also observed with *Pci*GLOX3 but not with *Pci*GLOX1.

**Fig. 6:**
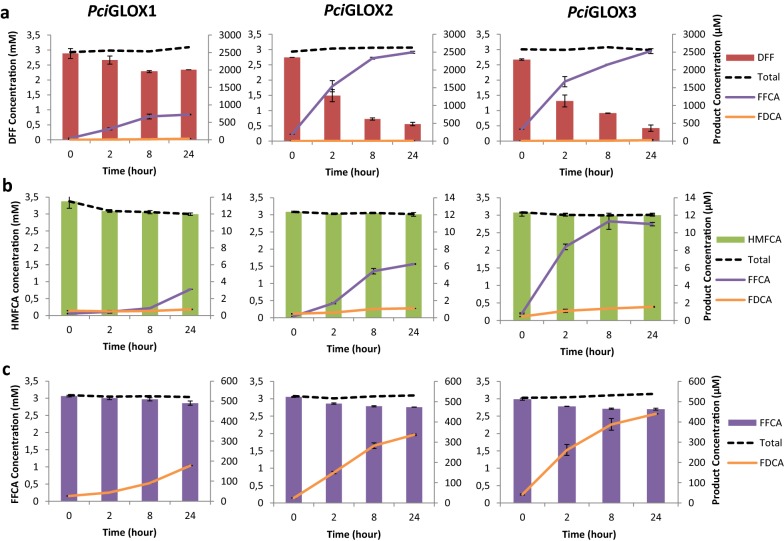
Follow-up of the reactions of *Pci*GLOX1, *Pci*GLOX2 and *Pci*GLOX3 on **a** DFF, **b** HMFCA and **c** FFCA as initial substrates. Bars represent standard deviation

Interestingly, small amounts of DFF, the alternative oxidation product of HMF, were also detected. The concentration of detected DFF slightly increased over time in the reaction with *Pci*GLOX1 (0.017 mM at 24 h). In the cases of *Pci*GLOX2 and *Pci*GLOX3, smaller DFF concentrations were detected early in the reaction. The substrate specificity and catalytic efficiencies of *Pci*GLOX2 and *Pci*GLOX3 on DFF as a substrate (*K*m = 0.2 mM; *K*cat/*K*m = 23,403 s^−1^ M^−1^ and *K*m = 0.2 mM; *K*cat/*K*m = 7267 s^−1^ M^−1^, respectively) were significantly better than that of *Pci*GLOX1 (*K*m = 4.3 mM; *K*cat/*K*m = 124.3 s^−1^ M^−1^). This was further supported in the results obtained when DFF was used as the initial substrate and 80% to 84% converted by *Pci*GLOX2 and *Pci*GLOX3, respectively (Fig. 6a).

The three *Pci*GLOX were also reacted with HMFCA as the initial substrate. Although this compound was produced as the major product in HMF oxidation, the three *Pci*GLOX showed very weak activity towards HMFCA, with *Pci*GLOX3 having the best conversion rate (2%; Fig. 6b). The extent of HMFCA to FFCA conversion by *Pci*GLOX was not sufficient to calculate catalytic constants. In addition, higher concentrations of FFCA and FDCA were obtained when the enzymes were reacted with DFF and FFCA as substrates, respectively (Fig. 6a, c).

### GLOX and AAO cascade reactions

**Fig. 7:**
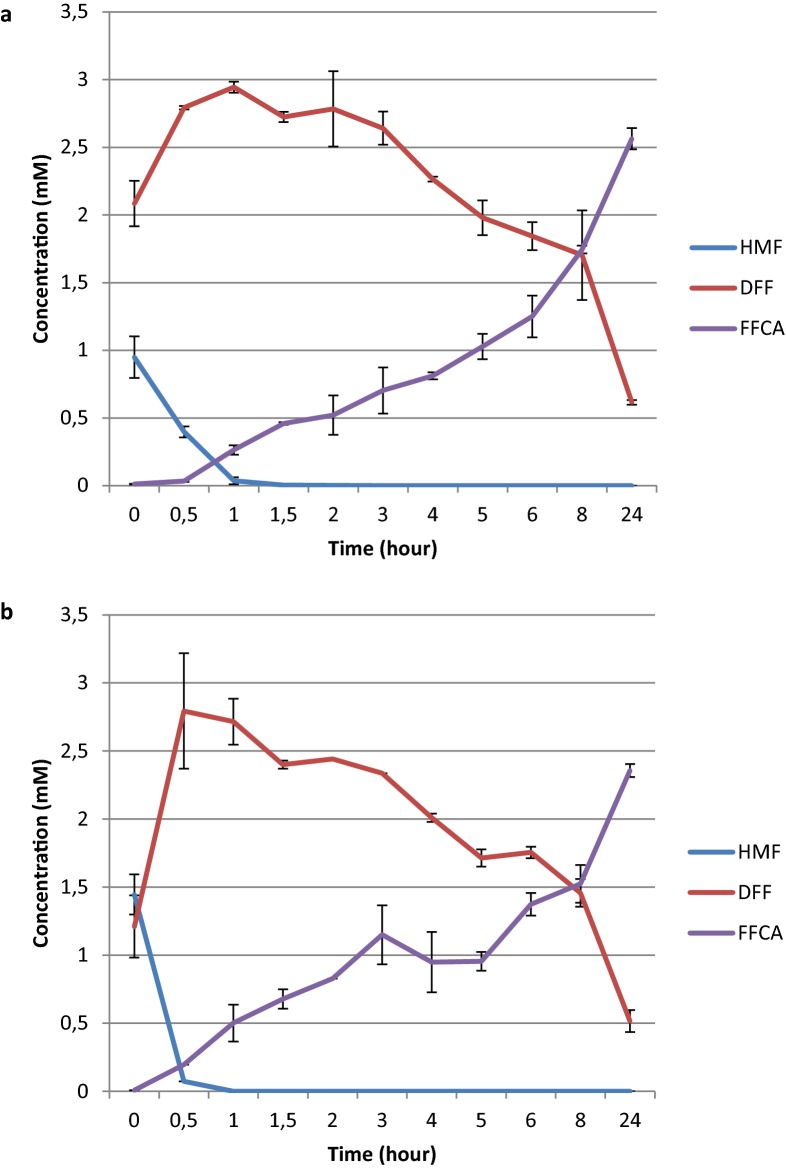
Oxidation of HMF overtime by *Uma*AAO in the presence (**a**) and absence (**b**) of catalase. Bars represent standard deviation

A cascade reaction where the aryl alcohol oxidase from *Ustilago maydis* (*Uma*AAO) was used to oxidize HMF followed by the addition of *Pci*GLOX was tested. *Uma*AAo alone was found to efficiently oxidize HMF leading to the formation to DFF as the major product after 2 h of reaction. DFF was further oxidized by *Uma*AAO forming FFCA after 24 h of reaction (Fig. 7; Additional file 2b). *Pci*GLOX3 was used in this experiment since this enzyme showed the highest conversion percentage on DFF (84% after 24 h) and the highest FDCA yields (14% after 24 h on FFCA). Two conditions were tested: (1) *Pci*GLOX3 was added after 2 h of *Uma*AAO reaction on HMF, and (2) *Pci*GLOX3 was added after 24 h of *Uma*AAO reaction on HMF. Under condition (1) HMF was completely oxidized by *Uma*AAO and the major product before the addition of *Pci*GLOX3 was DFF whereas in condition (2) FFCA was the predominant product.

After 4 h of adding *Pci*GLOX3, FFCA was the major product (2.6 mM) in the reaction and a little amount of DFF (0.5 mM) was detected (Table 4). Twenty hours later, DFF was almost completely consumed and the concentration of FDCA increased (0.03 mM). In the presence of catalase, DFF was completely oxidized after 4 h of reaction and the concentration of FDCA obtained was 6 folds higher after 24 h (Table 4). The yield of FDCA was further increased in condition (2) to reach 14% and 16% after 24 h in the absence and presence of catalase, respectively (Table 4). The addition of *Pci*GLOX3 to *Uma*AAO reaction on HMF was found to significantly increased the yields of FDCA (p = 0.026). The obtained yields in both tested conditions were comparable to the ones obtained when *Pci*GLOX3 was reacted with DFF and FFCA as the initial substrates (Fig. 6).

**Table 4:** Percentage content of HMF, DFF, FFCA and FDCA in the cascade reactions of *Uma*AAO and *Pci*GLOX3 following condition (1) that consisted of reacting *Uma*AAO with HMF for 2 h before adding *Pci*GLOX3 and condition (2) in which *Pci*GLOX3 was added after 24 h of the reaction of *Uma*AAO with HMF

	Condition (1)				Condition (2)			
	After 4 h		After 24 h		After 4 h		After 24 h	
	NC	C	NC	C	NC	C	NC	C
HMF	ND	ND^a^	ND	ND	ND	ND	ND	ND
DFF	17% ± 3.3	ND	1% ± 0.3	ND	ND	ND	ND	ND
FFCA	83% ± 1.2	96% ± 0.1	98% ± 0.7	94% ± 0.1	90% ± 6.2	87% ± 5.3	86% ± 0.4	84% ± 1
FDCA	ND	4% ± 0.8	1% ± 0.03	6% ± 0.03	10% ± 0.2	13% ± 0.6	14% ± 0.1	16% ± 1.2

Reactions were performed in the presence (C) and absence of catalase (NC) for both conditions. *Pci*GLOX3 was reacted for 4 or 24 h before analyzing the reaction products. Standard deviations are presented as plus-minus values

^a^ND, values below 1%

### Discussion

As mentioned in the introduction, HMF, a residue of the 2G lignocellulosic biorefinery, is of particular industrial importance due to its biodegradability and its versatility as a precursor for a wide selection of furan-based products, and as such has been recognized as a top value-added molecule in biotechnology. Currently, large chemical plants are being constructed to produce HMF, but a number of biological conversions have been identified in recent years, including the use of glyoxal oxidases as described in this paper. Although the three GLOX from *P. cinnabarinus* have a high protein sequence similarity and a conserved copper center, significant catalytic differences were observed and it was therefore interesting to compare the activity of all three enzymes on the conversion of HMF and its derivatives. The observed catalytic differences, in addition to the fact that *Pci*GLOX3 has not yet been found to be secreted during fungal growth on different substrates [28], suggests different physiological roles of these enzymes during fungal growth.

While enzymatic assays with *Pci*GLOX were performed at 30 °C, the temperature at which fungi usually grows and secretes these enzymes in vitro, the pH and buffer system were also previously found to be critical factors for activity [25]. *Pci*GLOX1 and *Pci*GLOX3 were very stable under the reaction conditions used, while *Pci*GLOX2 lost significantly activity over time, especially in the presence of its substrate, HMF. The stability of AAO from *P. eryngii*, which can also catalyze the oxidation of HMF to DFF, was previously determined in the presence of 3 mM HMF, and the enzyme was found to lose 30% of its activity after 24 h [18]. Similarly to HMF, the major reaction product HMFCA seems to alter considerably the catalytic properties of the *Pci*GLOX enzymes. It is difficult to determine if the observed changes in residual activity when HMF or HMFCA where added are due to changes in stability, or to either competitive or noncompetitive inhibition.

When incubated with different concentrations of H_2_O_2_ over time, the three isoenzymes again showed differences in stability. The inhibition of GLOX from *Phanerochaete chrysosporium* (*Pch*GLOX) by exogenous H_2_O_2_ has previously been reported [30]. However, the three *Pci*GLOX isoenzymes were found to be more stable than *Pch*GLOX, which retained only 25% of its initial activity in the presence of 2.1 mM exogenous H_2_O_2_ in the reaction mixture [30]. Furthermore, the detection of the reaction product of *Pch*GLOX on glycerol was only possible when the activity was extended by adding catalase [29], which is not the case for *Pci*GLOX [25]. H_2_O_2_ can oxidize proteins and alter the functional groups of certain amino acids leading to cleavage of the polypeptide chain and protein aggregation [31]. Therefore, one could postulate that the differences in stability observed between the three *Pci*GLOX in the presence of H_2_O_2_ could be explained by structural variations between the three isoenzymes.

Significant differences in the kinetic parameters and products’ yields for the oxidation of HMF were observed between the three enzymes, with the highest catalytic efficiency on HMF obtained with *Pci*GLOX3 (*K*cat/*K*m = 118.35 s^−1^ M^−1^). The oxidation of HMF using a phylogenetically different GLOX from *Myceliophthora thermophila* M77 (*Mt*GLOX) has been recently published [32]. Similarly to *Pci*GLOX described in this paper, low conversion levels were observed with HMF. However *Mt*GLOX showed a higher catalytic efficiency and specificity towards HMF (*K*cat/*K*m = 787 s^−1^ M^−1^, *K*m = 0.02 M^−1^) compared to *Pci*GLOX. Interestingly *Mt*GLOX oxidized the alcohol group of HMF leading to the formation of DFF.

Higher specificity and two-fold higher catalytic efficiency of AAO from *P. eryngii* were reported on HMF (*K*cat/*K*m = 210 s^−1^ M^−1^) compared to that recorded in this study on *Pci*GLOX [18]. In a more recent study using AAO for the oxidation of HMF, Karich and co-workers reported varying catalytic constants for the homologously produced AAO from *P. eryngii* on HMF with catalytic efficiency comparable to that obtained for *Pci*GLOX [33]. However, *P. eryngii* AAO was less specific on HMF. Another enzyme used for the oxidation of HMF is the FAD-dependent oxidase, HMFO, which was more specific on HMF compared to *Pci*GLOX and has a significantly higher catalytic efficiency (*K*cat/*K*m = 7000 s^−1^ M^−1^) [19]. A 95% conversion yield of HMF to FDCA was achieved using HMFO, however the concentration used was 60 times higher than the one used for *Pci*GLOX in this work which only requires oxygen as an electron acceptor.

The major product of the *Pci*GLOX reaction on HMF was HMFCA. This result was expected since GLOX recognizes the aldehyde group of its substrate [25], HMF in this case (Fig. 1). The need for this specificity has limited the number of described bio-catalytic pathways for the production of HMFCA. A previously described enzyme for this purpose is the bacterial xanthine oxidase (XO), which recognizes the formyl group of HMF [16]. Using 2.2 µM of this enzyme, HMFCA was obtained with a yield of 94% after 7 h of reaction on 26 mM HMF. The amount of *Pci*GLOX used in this work was comparable to XO, but the yield of HMFCA obtained with *Pci*GLOX2 after 24 h of reaction was significantly lower. However, a two folds increase in HMFCA yields was observed when catalase was added to *Pci*GLOX2 reaction. This was in agreement with the results showing that *Pci*GLOX2 was very sensitive to accumulating H_2_O_2_ concentrations. Catalase converts H_2_O_2_ to O_2_, protecting the enzymes from oxidative damage and supplying O_2_. This enzyme was previously used to eliminate the accumulating H_2_O_2_ from the action of alcohol oxidases on HMF [16]. In addition, catalase was found to boost HMF conversion in the coupled reaction of GAO and HRP, which is similar to the effect observed in this work [16].

In addition to HMFCA, small amounts of DFF were detected at early stages of the reaction especially with *Pci*GLOX1. This result shows that *Pci*GLOX are, to a smaller extent, able to act on the alcohol group of HMF. This is the second example of alcohol oxidation by these enzymes, which have been previously found to be active on glycerol [25, 29]. HMF oxidation to DFF as the only product was recently observed with *Mt*GLOX; however this enzyme was unable to further oxidize DFF in the reaction [32]. Enzymes known to convert HMF to DFF include also AAO, alcohol oxidase, GAO, and HMFO [16, 18, 19, 33, 34] and these enzymes showed higher yields of DFF compared to *Pci*GLOX. However, the substrate specificity and catalytic efficiencies of *Pci*GLOX2 and *Pci*GLOX3 on DFF as a substrate (*K*m = 0.2 mM; *K*cat/*K*m = 23,403 s^−1^ M^−1^ and *K*m = 0.2 mM; *K*cat/*K*m = 7267 s^−1^ M^−1^, respectively) were significantly better than that of AAO from *P. eryngii* (*K*m = 3.3 mM; *K*cat/*K*m = 158 s^−1^ M^−1^) and HMFO (*K*m = 1.7 mM; *K*cat/*K*m = 940 s^−1^ M^−1^) [18, 19]. *Pci*GLOX2 and *Pci*GLOX3 were also more efficient than *Pci*GLOX1 in oxidizing DFF which explains the presence of constant concentrations of DFF with *Pci*GLOX1. On the other hand, in the cases of *Pci*GLOX2 and *Pci*GLOX3, it is more likely that DFF was produced and consumed very fast in the reaction.

On the contrary, the major reaction product, HMFCA was very weakly oxidized by *Pci*GLOX. In addition, although the three enzymes and especially *Pci*GLOX2 and *Pci*GLOX3 were able to oxidize HMF, higher concentrations of FFCA and FDCA were obtained when the enzymes were reacted with DFF and FFCA as substrates, respectively.

In the reactions of *Pci*GLOX on HMF, very little DFF is produced compared to the “dead-end product” HMFCA which might be strongly altering the stability of the enzymes at early stages of the reaction preventing further oxidation steps. In addition, the obtained results suggest that similarly to AAO, HMFO and chloroperoxidase [18, 19, 34], *Pci*GLOX enzymes produce FFCA mainly via the oxidation of DFF (Fig. 1), which is a minor product in the oxidation of HMF by *Pci*GLOX. However, *Pci*GLOX are more specific and catalytically efficient than these enzymes in oxidizing DFF. For these reasons a cascade reaction, where *Uma*AAO was used to oxidize HMF followed by the addition of *Pci*GLOX, was tested. AAO was previously used in tandem reactions with an unspecific oxidase for the oxidation of HMF [18, 33]. Similarly to the AAOs from *Pleurotus eryngii* and *P. ostreatus*, *Uma*AAO generates FFCA via the oxidation of HMF to DFF [18, 33]. *Uma*AAO was also found as efficient as *P. eryngii* AAO in oxidizing HMF leading to full conversion after 2 h of reaction. In contrary to *P. eryngii* AAO, *Uma*AAO seems less efficient in converting DFF to FFCA in the reaction. However, the used concentration of *Uma*AAO in this study is significantly lower and a kinetic study is needed to determine the efficiency.

In the current *Pci*GLOX tandem reaction, the obtained yields of FDCA were lower compared to the ones obtained in the cascade reactions with UPO [18, 33]. A limiting step in the reaction of *Pci*GLOX seems to be the conversion of FFCA to FDCA and this was observed when FFCA was used as initial substrate. This effect is most possibly due to exogenous factors affecting the reaction since the enzymes exhibit relatively high specificity and catalytic efficiency towards FFCA. A probable factor could be the inhibition of the enzymes by the substrate and/or the product of the reaction. A second possible factor affecting this reaction is the accumulation of H_2_O_2_ throughout the first oxidation steps inhibiting the progress of the reaction in the FFCA oxidation stage. The sensitivity of *Pci*GLOX enzymes in the presence of H_2_O_2_ supports this hypothesis. It is also interesting that this effect was previously reported for *P. ostreatus* AAO where the sensitivity of FFCA oxidation reaction to H_2_O_2_ was 300-times higher than that of the initial reaction [33].

### Conclusion

The potential of *Pci*GLOX for the production of valuable furan derivatives from HMF was investigated in this work. The three GLOX belonging to the same organism and sharing high sequence similarity of 84–89%, showed differences in their catalytic properties and product patterns on the same substrates. Although known for their activity on aldehydes, these proteins were also able to act on the alcohol group of the substrate. Previously, this activity has been explained by the hypothesis that enzymes such as HMFO for example recognizes the hydrated form of the aldehyde in the reaction which explains their activity on DFF that rapidly forms hydrate in buffer pH 5–8 [19]. When the substrate has a carboxylic acid group in addition to the aldehyde group such as in the case of FFCA, the formation of hydrate is highly unfavorable and this explain the low activity of HMFO on FFCA [19, 35]. However, *Pci*GLOX were active on FFCA and were found to efficiently oxidize the aldehyde group of HMF. HMF was previously reported not to form gem-diol under conditions similar to the ones used in this work [18]. This shows that *Pci*GLOX are able to recognize and oxidize both aldehyde and, in certain cases, alcohol groups. HMFCA remains the major product of the reaction preventing the production of FDCA. However, the specificity of *Pci*GLOX towards producing HMFCA itself can be considered of high biotechnological significance. The addition of catalase, to remove the influence of self-inhibition through the production of H_2_O_2_, improved reaction yields, while the addition of an AAO prior to the addition of *Pci*GLOX shifted the reaction pathway through DFF and towards the production of FDCA, the desired precursor for bioplastic synthesis.

## Materials and methods

### Chemicals and enzymes

All chemicals were of analytical grade. HMF, DFF, HMFCA, FFCA, FDCA, 2,2′-azino-bis(3-ethylbenzothiazoline-6-sulphonic acid (ABTS) and glyoxylic acid, together with the enzymes HRP and catalase were purchased from Sigma Aldrich (Lyon, France). The glyoxal oxidases from *Pycnoporus cinnabarinus*, *Pci*GLOX1 and *Pci*GLOX2, were heterologously produced in *Aspergillus niger* as described previously [25]. The same protocol was used to produce the third isoform, *Pci*GLOX3, and is reported here for the first time. The aryl alcohol oxidase (AAO) from *Ustilago maydis* (*Uma*AAO) was produced as previously reported in our laboratory [36]. The sequences of *Pci*GLOX1, *Pci*GLOX2 and *Pci*GLOX3 are available in GenBank under accession numbers KU215437, KU215438 and MK268804, respectively.

### Enzyme activity assay

The activity of the three *Pci*GLOX enzymes was assayed in a coupled reaction with HRP following the protocol previously described [25]. When purified, GLOX enzymes are inactive and their oxidative activation was found to be possible in the presence of lignin peroxidase or HRP, or by the addition of strong oxidants such as molybdicyanide (K_3_Mo(CN)_8_), hexachloroiridate (Na_2_IrCl_6_), or Mn^3+^ EDTA [24, 37]. HRP was used in this study since it is readily obtained commercially, highly stable under the conditions used, and does not act on any of the substrates under investigation for oxidation by GLOX. The reaction mixture consisted of 50 mM sodium tartrate buffer (pH 6) containing HRP (8 Units), 0.1 mM ABTS, *Pci*GLOX (1 µg) and GLOX substrate at varying concentrations depending on the reaction in 1 mL final volume. The lag period was eliminated by adding 5 µM H_2_O_2_. The reaction was initiated by the addition of the GLOX substrate and the oxidation of ABTS was followed at 436 nm for 1.5 min. The standard assay was performed at 30 °C. All assays were performed in triplicate.

### *Pci*GLOX3 characterization

The substrate specificity of *Pci*GLOX3 on 10 mM of previously tested molecules (Table 1) with *Pci*GLOX1 and *Pci*GLOX2 was investigated following the activity test described above. The same standard assay was used to determine the kinetic constants for *Pci*GLOX3 on methylglyoxal (0.02 to 5 mM), glyoxal (0.1 to 7 mM), glyoxylic acid (0.1 to 10 mM), and glycerol (0.3 to 80 mM) as substrates. Lineweaver–Burk plots, obtained using the GraFit (version 4) program [38], were used to calculate the kinetic parameters.

The three *Pci*GLOX sequences were aligned using the ClustalW program within the MegAlign module (version 11.0.0) of DNAStar software (Madison, WI).

### Enzyme stability

The three *Pci*GLOX were incubated at 30 °C and 800 rpm agitation for 48 h in 50 mM sodium tartrate buffer pH 6. Samples were taken after 2, 4, 24 and 48 h and the residual activity was measured using glyoxylic acid as substrate and calculated as a percentage of the activity at time zero. The same experiment was also performed in the presence of 3 mM HMF or HMFCA to determine the stability of the enzymes in the presence of these molecules. The stability of HRP was also assessed under the same conditions. All measurements were performed in triplicates.

### Kinetic studies

The coupled reaction with HRP and ABTS under standard conditions was used to determine the steady-state kinetic parameters for *Pci*GLOX oxidation of HMF (0.3–50 mM), DFF (0.01–2 mM), HMFCA (0.1–3 mM) and FFCA (0.2–5 mM). Kinetic parameters were obtained by fitting the data to the Michaelis–Menten equation using R statistical software (R Core Team, Vienna Austria).

### HPLC analysis of reaction products

All oxidation reactions were performed at 30 °C under agitation at 800 rpm in Eppendorf ThermoMixer tubes (Eppendorf, Montesson, France) over 24 h period in 100 mM tartrate buffer, pH 6. To follow the reaction of the three *Pci*GLOX isoenzymes on HMF, DFF, HMFCA and FFCA over time, reaction mixtures containing *Pci*GLOX (20 µg), HRP (8 Units), ABTS (0.1 mM) and the substrate (3 mM) were prepared. Samples were taken after 0, 0.5, 1, 1.5, 2, 3, 4, 5, 6, 8 and 24 h and analyzed. Reactions containing the same constituents except *Pci*GLOX were followed for the same period of time and used as controls. All measurements were performed in duplicates.

Reaction mixtures were separated on Aminex HPX-87H column (300 × 7.8 mm) (BioRad) at 45 °C, with 2.5 mM sulfuric acid as the mobile phase with a flow rate of 0.6 mL/min. Eluted compounds were detected using a diode array detector at 280 nm. The reactions were stopped by incubating the mixture at 90 °C for 10 min and centrifuging at 15,000×*g* for 15 min. The samples were then filtered using 0.45 µm polyvinylidene difluoride syringe filters (Restek, Lisses, France) before injection in the column. Peak areas from the obtained chromatograms were converted to molar concentration using calibration curves of pure substrates and products standards.

The percentage conversion of the used substrate was determined according to the following equation:

$$\% Conversion = \left( {1{-}\frac{Concentration\;of\;substrate}{Initial\;concentration\;of\;substrate}} \right) \times 100$$

The percentage yield of the reaction products was determined according to the following equation:

$$\% Yield = \left( {\frac{Concentration\;of\;product}{Initial\;concentration\;of\;substrate}} \right) \times 100$$

### Effect of H_2_O_2_ on HMF oxidation

The stability of the three *Pci*GLOX isoenzymes in the presence of H_2_O_2_ was assessed by measuring the residual activity of the enzymes after pre-incubation with different concentrations of H_2_O_2_ over 24 h. The H_2_O_2_ was removed before adding the enzyme to the reaction mixture by washing the samples with buffer in Amicon ultrafiltration unit with a 10-kDa-molecular-mass-cut-off membrane (Merck Millipore). All measurements were performed in duplicate.

To determine the effect of hydrogen peroxide on the oxidation of HMF and its derivatives by *Pci*GLOX, the standard reaction was performed in the presence of 10 µg catalase (2000–5000 Units/mg protein). The reaction was followed over time as described above. A control reaction containing all the components except *Pci*GLOX was analyzed to determine the effect of catalase on HMF oxidation.

### *Pci*GLOX and *Uma*AAO cascade reactions

The oxidation of HMF by *Uma*AAO in the presence and absence of 10 µg catalase was followed. The reaction mixture contained *Uma*AAO (20 µg) and HMF (3 mM) in 100 mM tartrate buffer, pH 6. Samples were taken after 0, 0.5, 1, 1.5, 2, 3, 4, 5, 6, 8 and 24 h and analyzed on HPLC. All measurements were performed in duplicates.

*Uma*AAO was then used in a cascade reaction with *Pci*GLOX3 for the oxidation of HMF and its derivatives. The reaction was performed by reacting *Uma*AAO with HMF for 2 or 24 h before adding *Pci*GLOX3. Following the addition of *Pci*GLOX3, the samples were further incubated for 4 or 24 h and then analyzed. The reactions were again performed in the presence and absence of catalase.

### Statistical analysis

The statistical analyses of data on the effect of catalase on the activity of *Pci*GLOX on HMF and the effect of *Pci*GLOX3 on the production of FDCA in the cascade reaction with *Uma*AAO were performed by the non-parametric Kruskal–Wallis test (Kruskal & Wallis, 1952; p < 0.05). The analysis was followed by post hoc Dunn pairwise comparison test (Dunn 1964) when differences were significant.

## Additional files

**Additional file 1.** Stability of HRP under the reaction conditions. Residual activity of HRP on ABTS after incubation for different time periods (0–24 h) in tartrate buffer pH 6 at 30 °C and 800 rpm (back) and in the presence of 3 mM HMF (blue) or HMFCA (green).

**Additional file 2.** HPLC chromatogram of the reactions of PciGLOX and UmaAAO on HMF. HPLC chromatogram of the reactions of (a) *Pci*GLOX1 (red), *Pci*GLOX2 (green) and *Pci*GLOX3 (pink) enzymes on HMF after 24 h of incubation compared to the control (blue) and (b) *Uma*AAO on HMF at t_0_ (blue) and after 1 h (red), 2 h (green), 4 h (pink) and 24 h (grey) of reaction.

## Abbreviations

HMF: 5-hydroxymethylfurfural
FDCA: 2,5-furandicarboxylic acid
DFF: diformylfuran
HMFCA: 5-hydroxymethyl-2-furancarboxylic acid
GAO: galactose oxidase
AAO: aryl alcohol oxidase
HMFO: HMF oxidase
GLOX: glyoxal oxidase
FFCA: 5-formyl-2-furan carboxylic acid
ABTS: 2,2′-azino-bis(3-ethylbenzothiazoline-6-sulphonic acid
XO: xanthine oxidase
HRP: horseradish peroxidase
