# Supplementary figures and images for: Pycnoporus cinnabarinus glyoxal oxidases display differential catalytic efficiencies on 5-hydroxymethylfurfural and its oxidized derivatives
Source: Fungal Biol Biotechnol. 2019 Apr 1;6:4. doi: 10.1186/s40694-019-0067-8 (PMC6442418; doi:10.1186/s40694-019-0067-8)

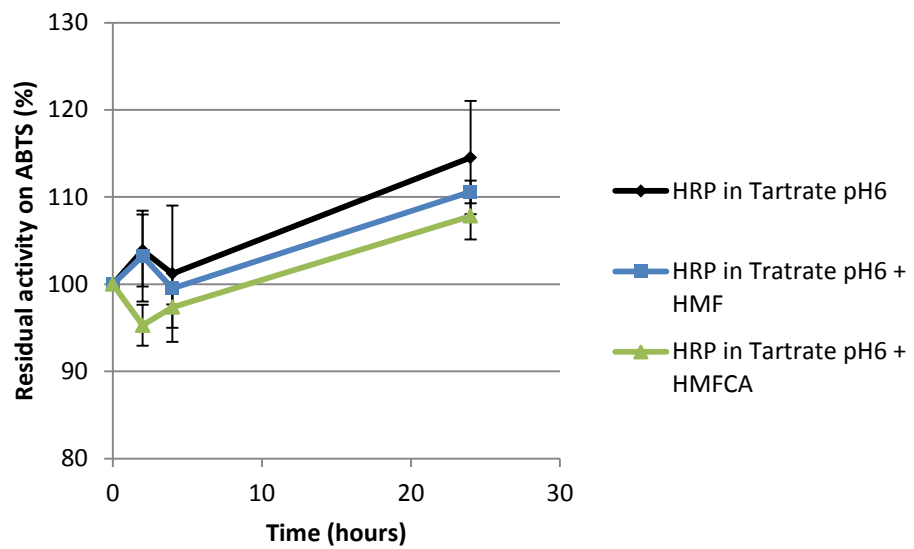

Supplement: Supplementary file 1 — Additional file 1. Stability of HRP under the reaction conditions. Residual activity of HRP on ABTS after incubation for different time periods (0–24 h) in tartrate buffer pH 6 at 30 °C and 800 rpm (back) and in the presence of 3 mM HMF (blue) or HMFCA (green). [file 40694_2019_67_MOESM1_ESM.pdf]

a.

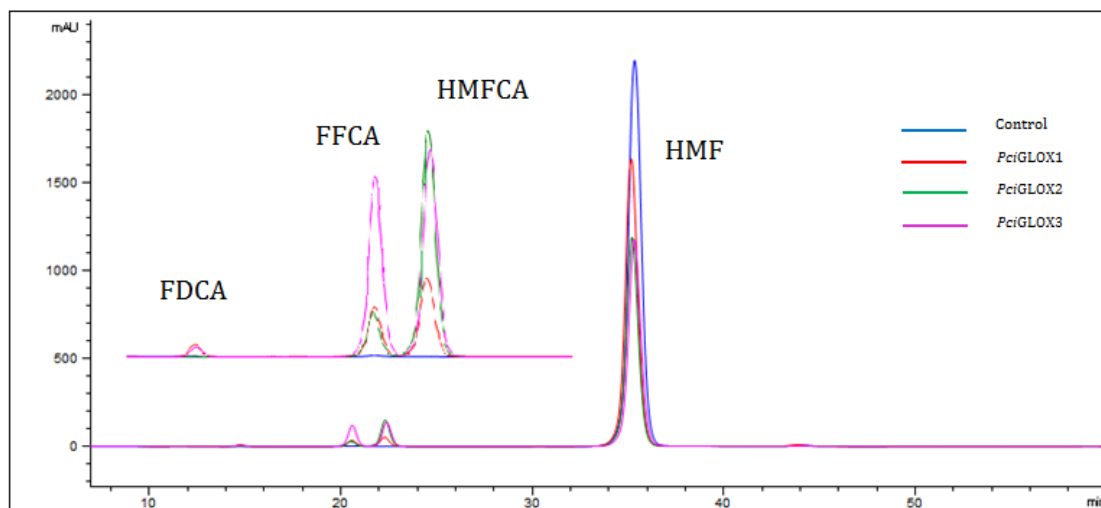

b.

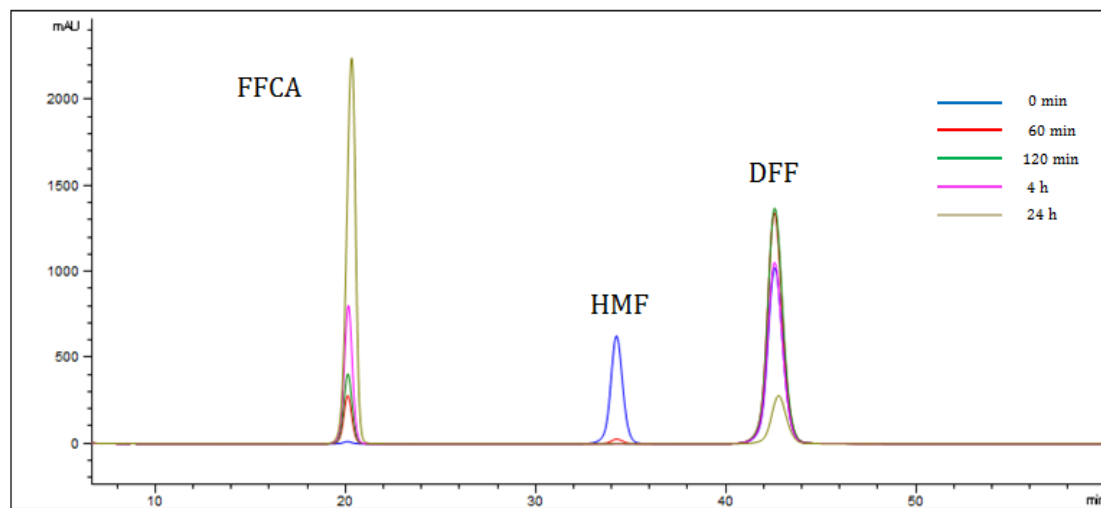

Supplement: Supplementary file 2 — Additional file 2. HPLC chromatogram of the reactions of PciGLOX and UmaAAO on HMF. HPLC chromatogram of the reactions of (a) PciGLOX1 (red), PciGLOX2 (green) and PciGLOX3 (pink) enzymes on HMF after 24 h of incubation compared to the control (blue) and (b) UmaAAO on HMF at t0 (blue) and after 1 h (red), 2 h (green), 4 h (pink) and 24 h (grey) of reaction. [file 40694_2019_67_MOESM2_ESM.pdf]
